# Purposive sampling in a qualitative evidence synthesis: a worked example from a synthesis on parental perceptions of vaccination communication

**DOI:** 10.1186/s12874-019-0665-4

**Published:** 2019-01-31

**Authors:** Heather Ames, Claire Glenton, Simon Lewin

**Affiliations:** 1 Cochrane Consumers and Communication Group, La Trobe University, Melbourne, Australia; 2 Division for Health Services, Norwegian Institute of Public Health, Postboks 222 Skøyen, Sandakerveien 24C, inngang D11, 0213 Oslo, Norway; 3 Cochrane Norway and the Informed Health Choices Research Centre, Norwegian Institute of Public Health, Postboks 222 Skøyen, Sandakerveien 24C, inngang D11, 0213 Oslo, Norway; 4 Cochrane EPOC group and the Informed Health Choices Research Centre, Norwegian Institute of Public Health, Postboks 222 Skøyen, Sandakerveien 24C, inngang D11, 0213 Oslo, Norway; 5 Health Systems Research Unit, South African Medical Research Council, Tygerberg, South Africa

**Keywords:** Purposive sampling, Qualitative evidence synthesis, Systematic review

## Abstract

**Background:**

In a qualitative evidence synthesis, too much data due to a large number of studies can undermine our ability to perform a thorough analysis. Purposive sampling of primary studies for inclusion in the synthesis is one way of achieving a manageable amount of data. The objective of this article is to describe the development and application of a sampling framework for a qualitative evidence synthesis on vaccination communication.

**Methods:**

We developed and applied a three-step framework to sample studies from among those eligible for inclusion in our synthesis. We aimed to prioritise studies that were from a range of settings, were as relevant as possible to the review, and had rich data. We extracted information from each study about country and study setting, vaccine, data richness, and study objectives and applied the following sampling framework:

1. Studies conducted in low and middle income settings
2. Studies scoring four or more on a 5-point scale of data richness
3. Studies where the study objectives closely matched our synthesis objectives

**Results:**

We assessed 79 studies as eligible for inclusion in the synthesis and sampled 38 of these. First, we sampled all nine studies that were from low and middle-income countries. These studies contributed to the least number of findings. We then sampled an additional 24 studies that scored high for data richness. These studies contributed to a larger number of findings. Finally, we sampled an additional five studies that most closely matched our synthesis objectives. These contributed to a large number of findings.

**Conclusions:**

Our approach to purposive sampling helped ensure that we included studies representing a wide geographic spread, rich data and a focus that closely resembled our synthesis objective. It is possible that we may have overlooked primary studies that did not meet our sampling criteria but would have contributed to the synthesis. For example, two studies on migration and access to health services did not meet the sampling criteria but might have contributed to strengthening at least one finding. We need methods to cross-check for under-represented themes.

**Electronic supplementary material:**

The online version of this article (10.1186/s12874-019-0665-4) contains supplementary material, which is available to authorized users.

## Background

Qualitative evidence syntheses, also known as systematic reviews of qualitative research, aim to explore people’s perceptions and experiences of the world around them by synthesizing data from studies across a range of settings. When well-conducted, a qualitative evidence synthesis provides an in-depth understanding of complex phenomena while focusing on the experiences and perceptions of research participants and taking into consideration other contextual factors [1]. Qualitative evidence synthesis first appeared as a methodology in the health sciences in the mid-1990s [2]. The approach is still relatively rare compared to systematic reviews of intervention effectiveness, but is becoming more common [3], and organisations such as Cochrane are now undertaking these types of synthesis [4–6]. The ways in which these syntheses are conducted has evolved over the last 20 years and now includes a variety of approaches such as meta-ethnography, thematic analysis, narrative synthesis and realist synthesis [2, 7].

For some qualitative evidence synthesis questions, there are a large number of primary qualitative studies available, and there are several examples of syntheses that include more than 50 studies [8]. However, in contrast to reviews of effectiveness, the inclusion of a large number of primary studies with a high volume of data is not necessarily viewed as an advantage as it can threaten the quality of the synthesis. There are a number of reasons for this: firstly, analysis of qualitative data requires a detailed engagement with text. However, large volumes of data make this difficult to achieve, and can make it difficult to move from descriptive or aggregative analysis to more interpretive analysis. Similar to the argument made for primary qualitative research [9, 10], the more data a researcher has to synthesize, the less depth and richness they are likely to be able to extract from the data. Furthermore, effectiveness reviews aim to be exhaustive in order to achieve statistical generalizability which requires certain procedures whereas qualitative evidence synthesis aim to understand the phenomenon of interest and how it plays out in a context. This requires gathering data from the various contexts and respondent groups relevant to understanding the phenomenon. This is done in a purposeful way to gather data relevant to answering the review question. Exhaustive searching and inclusion can undermine this understanding, as qualitative synthesis seek to achieve conceptual and not statistical generalizability.

The sampling of studies within qualitative evidence syntheses is still a relatively new methodological strategy, but is generally based on the same principles as those used to conduct sampling within primary qualitative research [11, 12]. There has been little written on how best to limit the number of included studies in a qualitative evidence synthesis and there is currently no agreement amongst review authors and methodologists about the best approach [13]. Options include sampling from the range of eligible studies (similar to purposively sampling participants within primary qualitative research) or narrowing the scope of the research question by, for example, geographic area or population. Suri [14] proposes a range of different strategies that could be applied to purposively sample for a qualitative evidence synthesis (see Table 1 for examples). These methods are adapted from a list by Patton for primary research purposes [12]. A recent paper by Benoot, Hannes et al. gives a worked example of sampling for a qualitative evidence synthesis [15]. However, there are few other well-described examples of the use of these approaches and it is not yet clear which approaches are best suited to particular kinds of synthesis, synthesis processes and questions.

**Table 1:** Some examples of purposeful sampling methods [14]

Type of sampling	Description
Extreme or deviant case sampling	• Selecting illuminative cases that exemplify ‘extreme’ or ‘deviant’ contexts or examples, for instance: – where an innovation in a primary study was perceived notably as a success or failure – where findings of a primary study are very different from those of most studies identified for the synthesis
Maximum variation sampling	• Constructed by: – identifying key dimensions of variation, and then – finding cases that vary from each other as much as possible along these dimensions • This sampling yields: – ‘high-quality, detailed descriptions of each case, which are useful for documenting uniqueness, and – important shared patterns that cut across cases and derive their significance from having emerged out of heterogeneity’ (Patton, 2002, p. 235)
Snowball or chain sampling	• Trying to locate a key work in the field through talking with experts or locating a key article that is often cited • Then follow on with primary studies that have cited the key or landmark study
Theoretical or operational construct sampling	• Selecting cases that represent important theoretical or operational constructs about the phenomenon of interest • Set out operational definitions of key theories or constructs related to the phenomenon of interest • Develop boundaries for these by creating specific inclusion and exclusion criteria in relation to selecting primary studies for the synthesis
Criterion sampling	• Used by those trying to construct a comprehensive understanding • Studies are sampled based on a predetermined criteria • Specific inclusion and exclusion criteria are clearly stated • Studies are then analysed as a whole
Stratified purposeful sampling	• Following on from criterion sampling where each of the criteria would become a sample • Stratified samples are samples within samples where each stratum, or group, is fairly homogenous and are analysed within these groups • Useful for examining variation in a key phenomena of interest
Purposeful random sampling	• Randomly select from the list of included studies for inclusion in the analysis • For example, use a random internet based selector, choose every 3rd included study or pull study names from a hat • Provides an unbiased way of selecting studies for inclusion but may not provide studies with rich data
Combination or mixed purposeful sampling	• Choosing a combination or mix of sampling strategies to best fit your purpose – For some syntheses, it may be useful to use a combination or mix of sampling strategies. For instance, by applying theoretical sampling in a first stage and deviant case sampling in a second stage. This should be guided by the review methods and purpose, and the time available

The example of sampling for a qualitative evidence synthesis presented in this article is drawn from a Cochrane qualitative evidence synthesis on parents’ and informal caregivers’ views and experiences of communication about routine childhood vaccination [5]. We understood at an early stage that the number of studies eligible for this synthesis would be high. As there was limited guidance on how to sample studies for inclusion in a qualitative evidence synthesis, we had to explore ways of solving this methodological challenge. The objective of this paper is to discuss the development and application of a sampling framework for a qualitative evidence synthesis on vaccination communication and the lessons learnt.

## Methods

The objective of our qualitative evidence synthesis was to identify, appraise and synthesise qualitative studies exploring parents’ and informal caregivers’ views and experiences regarding the communication they receive about childhood vaccinations and the manner in which they receive it [5]. To be eligible for inclusion in the synthesis, studies had to have used qualitative methods of data collection and analysis; had parents or informal caregivers as participants; and had a focus on views and experiences of information about childhood vaccination. In August 2016, we searched MEDLINE, Embase, CINAHL and Anthropology Plus for eligible studies. We chose these databases as we anticipated that they would provide the highest yield of results based on preliminary, exploratory searches [5].

Seventy-nine studies met our eligibility criteria. We decided that this number of included studies was too large to analyse adequately and discussed whether it would be reasonable to limit our synthesis to specific settings or certain types of childhood vaccines. However, we concluded that narrowing the scope of the synthesis was not an acceptable option as we were interested in identifying global patterns concerning parental preferences for information. We mapped the eligible studies by extracting key information from each study, including information about country, study setting, vaccine type, participants, research methods and study objectives. This mapping of the included studies also showed that it would be difficult to narrow by vaccine type as the majority of the studies did not state explicitly which vaccines the study encompassed but focused instead on parents’ and caregivers’ views on childhood vaccination communication in general. We therefore decided to sample from the included studies.

Our main aim when sampling studies was to protect the quality of our analysis by ensuring that the amount of data was manageable. However, we also wanted to ensure that the studies we sampled were the most suitable for answering our objectives. As this was a global review, we were looking for studies that covered a broad range of settings, including high, middle and low income countries. In addition, we wanted studies that were as close as possible to the topic of our synthesis and that had as rich data as possible.

When considering how to achieve these goals, we assessed all of the 16 purposeful sampling methods proposed in the Suri study [14]. However, none of these directly fit all of our needs although some of the methods addressed some of these needs (See Table 6). We therefore reshaped the approaches described in Suri, combining different sampling strategies to create our own purposive sampling framework, as has been done by others [15].

We developed the sampling framework taking into consideration the data that had been mapped from the included studies and what would best fit with our research objective. The sampling framework was piloted on a group of ten studies and the review authors discussed challenges that arose. Our final, three-step sampling framework was as follows:

### Step 1: Sampling for maximum variation

Our focus was to develop a global understanding of the phenomenon of interest, including similarities and differences across different settings. The majority of the studies that met the inclusion criteria took place in high-income settings. Our first step was therefore to sample all studies from low and middle-income countries. This helped us to ensure a geographic spread and reasonable representation of findings from all income settings. The inclusion of these studies was also important because of the interest globally in improving vaccination uptake in these settings, and this was also part of the ‘Communicate to vaccinate’ project in which the synthesis was embedded [16].

### Step 2: Sampling for data richness

Second, to ensure that we would have enough data for our synthesis, we focused on the richness of the data within the remaining included studies. We based this decision on the rationale that rich data can provide in-depth insights into the phenomenon of interest, allowing the researcher to better interpret the meaning and context of findings presented in the primary studies [17]. To our knowledge there is no existing tool to map data richness in qualitative studies. We therefore created a simple 1–5 scale for assessing data richness (see Table 2). After assessing the data richness of the remaining included studies, we sampled all studies that scored a 4 or higher for data richness.

**Table 2:** Data richness scale used during sampling for Ames 2017 [5]

Score	Measure	Example
1	Very few qualitative data presented. Those findings that are presented are fairly descriptive	For example, a mixed methods study using open ended survey questions or a more detailed qualitative study where only part of the data relates to the synthesis objective
2	Some qualitative data presented	For example, a limited number of qualitative findings from a mixed methods or qualitative study
3	A reasonable amount of qualitative data	For example, a typical qualitative research article in a journal with a smaller word limit and often using simple thematic analysis
4	A good amount and depth of qualitative data	For example, a qualitative research article in a journal with a larger word count that includes more context and setting descriptions and a more in-depth presentation of the findings
5	A large amount and depth of qualitative data	For example, from a detailed ethnography or a published qualitative article

### Step 3: Sampling for study scope / sampling for match of scope

Finally, we anticipated that studies that closely matched our objectives were likely to include data that was most valuable for the synthesis, even if those data were not very rich. After applying the first two sampling steps, we therefore examined the studies that remained and sampled studies where the study findings and objectives most closely matched our synthesis objectives. Studies were eligible for inclusion in the synthesis if they included at least one theme regarding parental perceptions about vaccination communication. However, many of these studies focused on parental perceptions of vaccination or vaccination programs rather than on parental perceptions of vaccination communication more specifically. In this final sampling step, we looked for studies that had *primarily* focused on parental perceptions about vaccination information and communication but had not been sampled in the first two steps. For example, an article exploring what informs parents’ decision making about childhood vaccination [18] was not included in step 1 as it was not from a low or middle income country or in step 2 as it scored a 3 for data richness. It was sampled in step 3 as its focus on information closely matched to the synthesis objectives.

We listed studies that met our inclusion criteria but were not sampled into the analysis in a table in the published qualitative evidence synthesis. The table provided the reason why the study was not sampled. This table provides readers with an overview of the existing research literature, makes our decision making process transparent and allows readers to critically appraise our decisions.

After the qualitative evidence synthesis was completed, we mapped the step during which each study was sampled and the number of findings to which each study had contributed. (See Appendix 1) We did this to see if the step at which the study was sampled into the review had an impact on the number of findings it contributed to; allowing us to see if studies sampled for richer data or closeness to the review objective did actually contribute to more findings.

During the process of writing the qualitative evidence synthesis, the review authors continued to discuss the strengths and weaknesses of the approach used to identify the issues presented in this paper. We also presented the approach to other teams doing qualitative evidence syntheses, and at conferences and meetings. These presentations and ensuing discussions facilitated the identification of other strengths and weaknesses of the approach that we had used. (See Table 6).

## Results

Seventy-nine studies were eligible for inclusion in the synthesis. After applying our sampling framework, we included thirty-eight studies.

### Our experiences when applying the sampling framework

The sampling approach we used in this review aimed to achieve a range of settings, studies with rich data and studies with findings that matched our review objective. We aimed to build a sampling framework that specifically addressed and was in harmony with the synthesis objectives.

### Sampling means that we may miss articles with information about particular populations, settings, or interventions

One of the main challenges of using a sampling approach is that we are likely to have omitted data related to particular populations, settings, communication strategies, vaccines or experiences. However, we argue that this approach allowed us to achieve a good balance between the quality of the analysis and the range of settings and populations within the included studies. First we will present a challenge related to setting and second a challenge related to population.

The first challenge we addressed was related to study setting. Our sampling approach did not directly select studies conducted in high income countries, and this led to some studies from these settings not being sampled. However, we decided that geographic spread was an important factor for this global synthesis and sampled accordingly. This is a limitation of our sampling frame. However, we believe that it was a strength to have studies from a wider variety of settings to increase the relevance of the findings to a larger number of contexts.

The second challenge relates to study population. Our sampling frame did not directly sample for variation in study populations. One clear example of how studies were missed that could have directly contributed to a finding related to a specific study population came with the issue of migration and vaccination.

Finding 6: Parents who had migrated to a new country had difficulty negotiating the new health system and accessing and understanding vaccination information.

We did not sample a few primary studies that discussed migrant issues specifically, as they did not meet the sampling criteria; specifically, they were not from LMIC contexts, had thin data or did not closely match the synthesis objectives. They most likely would have contributed to strengthening at least the finding described above.

### Our sampling framework meant that we may have sampled studies with thinner data

With our decision to focus on study location in step 1 of our sampling we may have sampled studies from low and middle-income contexts that scored a 1 or 2 for data richness (a potential weakness) and not sampled studies from high income settings with richer data. We were unsure whether the amount of relevant data in the studies from low and middle-income settings would make a contribution to the synthesis and findings. In the end we decided to include these studies to address the issue of relevance for LMIC contexts since the synthesis had a global perspective. However, this meant that studies with richer data from more privileged settings were not sampled. To adjust for this the second step of sampling was directly linked to data richness. All studies scoring a 4 or higher for data richness were sampled.

### Making decisions on how to assess data richness?

Initially, we looked at the whole study when assessing data richness. However, we realised that much of this data covered topics that were outside of the scope of the synthesis. This included, for example, information on parents perceptions of vaccines in general, advice they had received from unofficial sources such as friends and neighbours and their thoughts about how susceptible their children were to vaccine preventable diseases.

We therefore adapted the data richness scale to combine steps 2 and 3 of our sampling framework. The end result was a table where the richness of data in an included study is not ranked by the total amount of data but by the amount of data that is relevant to the synthesis objectives (see Table 3). This approach has since been used successfully in a new synthesis (Ames HMR, Glenton C, Lewin S, Tamrat T, Akama E, Leon N: Patients and peoples’ perceptions and experiences of targeted digital communication accessible via mobile devices for reproductive, maternal, newborn, child and adolescent health: a qualitative evidence synthesis. Submitted).

**Table 3:** Revised data richness table

Score	Measure	Example
1	Very little qualitative data presented that relate to the synthesis objective. Those findings that are presented are fairly descriptive.	For example, a mixed methods study using open ended survey questions or a more detailed qualitative study where only part of the data relates to the synthesis objective
2	Some qualitative data presented that relate to the synthesis objective	For example, a limited number of qualitative findings from a mixed methods or qualitative study
3	A reasonable amount of qualitative data that relate to the synthesis objective	For example, a typical qualitative research article in a journal with a smaller word limit and often using simple thematic analysis
4	A good amount and depth of qualitative data that relate to the synthesis objective	For example, a qualitative research article in a journal with a larger word count that includes more context and setting descriptions and a more in-depth presentation of the findings
5	A large amount and depth of qualitative data that relate in depth to the synthesis objective.	For example, from a detailed ethnography or a published qualitative article with the same objectives as the synthesis

### Did the sampling step impact on how many findings a study contributed to?

It has been suggested that studies with richer data, also described as conceptual clarity, may self-weight in the findings of qualitative evidence syntheses (contribute more data to the synthesis) and be found to be more methodologically sound [19, 20]. In order to test this we mapped the step in which the studies were sampled and the number of findings each study contributed to. The rationale for this was that we sampled studies that had a lower score for data richness in steps one and three. If these studies contributed to a distinctly lower number of study findings this could reinforce the idea that studies with richer data (i.e. step two) contributed more data to more findings than studies with thinner data. To some extent this was the case with the studies sampled in step one from low and middle-income contexts. However, this did not apply as well to studies sampled in step three where the study findings were more closely aligned with the synthesis objectives. (See Table 4).

**Table 4:** Overview of sampling stage and contribution to findings for primary studies included in the Qualitative Evidence Synthesis

Sampling step	Number of studies that were sampled	Average and range of number of findings that these studies contributed to
1- LMIC settings	9	6 (2–13)
2– Score of three or more for data richness	24	13 (3–20)
3– Closeness to the synthesis objectives	5	13 (6–22)

Nine studies from LMIC contexts were sampled in step one and these contributed to, on average, the least number of synthesis findings. Twenty-four studies were sampled on the basis of data richness in step two; these contributed to a large number of findings. The five studies sampled in step three because their findings most closely matched the synthesis objectives also contributed to a large number of findings. Table 4 shows the overview of how many studies were sampled in each step and how many findings the studies contributed to (See additional file 1 for a detailed overview per study).

## Discussion

We believe that our sampling framework allowed us to limit the number of studies included in the synthesis in order to make analysis manageable, while still allowing us to achieve the objectives of the synthesis.

The decision to purposively sample primary studies for inclusion in the qualitative evidence synthesis had its strengths and weaknesses. It allowed us to achieve a sufficiently wide geographic spread of primary studies while limiting the number of studies included in the synthesis. It enabled us to include studies with rich data and studies that most closely resembled the synthesis objectives. However, we may have overlooked primary studies that did not meet the sampling criteria but would have contributed to the synthesis. Furthermore, this qualitative evidence synthesis used a thematic approach to synthesis. Different synthesis approaches may have led us towards different ways of sampling or have identified different findings.

The approach for assessing richness of data needs to be developed further and tested within other qualitative evidence syntheses to see if it needs adjustment. It has worked well for the two syntheses we have used it in and has been understandable to other authors as a logical tool for mapping how much relevant data is in each included study [21] (Ames HL N, Glenton C, Tamrat T, Lewin S: Patients’ and clients’ perceptions and experiences of targeted digital communication accessible via mobile devices for reproductive, maternal, newborn, child and adolescent health: a qualitative evidence synthesis (protocol), unpublished) . However, objective testing of the scale would be needed to assess its validity across research teams and to standardize its approach.

### Working with the GRADE-CERQual approach to develop sampling for qualitative evidence synthesis in the future

Qualitative evidence syntheses are increasingly using GRADE-CERQual (hereafter referred to as CERQual) to assess the confidence in their findings. CERQual aims to transparently assess and describe how much confidence decision makers and other users can place in individual synthesis findings from syntheses of qualitative evidence. Confidence in the evidence has been defined as an assessment of the extent to which the synthesis finding is a reasonable representation of the phenomenon of interest. CERQual includes four components [22, 23] (Table 5).

**Table 5:** The four components of CERQual [23, 7]

Methodological limitations	The extent to which there are problems in the design or conduct of the primary studies that contributed evidence to a synthesis finding
Coherence	The extent to which the synthesis finding is well grounded in data from the contributing primary studies and provides a convincing explanation for the patterns found in the data
Adequacy of data	An overall determination of the degree of richness and quantity of data supporting a synthesis finding
Relevance	The extent to which the body of evidence from the primary studies supporting a synthesis findings is applicable to the context (perspective or population, phenomenon of interest, setting) specified in the synthesis question

We believe that purposive sampling would be useful to address concerns that arise during the CERqual process, specifically regarding relevance and adequacy. However, all four components could be taken into consideration when developing a sampling frame.

#### Relevance

Relevance addresses a number of study characteristics (see Additional file 2). It links to the approach we took in step 1 to include a maximum variation of settings. Review authors could use the relevance concept to design their sampling framework to address key study characteristics. A review author could also return to the pool of included studies and sample studies that would help to moderate downgrading in relation to these concepts. For example, if a synthesis finding was downgraded for relevance as all of the studies were conducted in a specific context or geographic location the authors could go back and sample studies from other contexts to address relevance concerns.

#### Adequacy

The adequacy component of CERQual links to our assessment of data richness. Is there enough data and rich data to support a synthesis finding? By sampling studies with richer data we believe that adequacy could be improved.

Related to the concepts of data richness and adequacy of data is the concept of data saturation. Our aim was not to reach data saturation for each of the findings in the synthesis through sampling. It would be possible to develop a sampling approach geared towards the concept of saturation however, this would be different from completing sampling before the analysis stage of the synthesis. If you were to sample with the aim of saturation it would be natural to sample from your included primary studies during the analysis process, in a sequential way.

#### Methodological limitations

A potential weakness of our approach is that we did not sample studies based on their methodological limitations. This means that primary studies that were methodologically weak may have been included in the synthesis if they met our sampling criteria. This has implications for our CERQual assessment of confidence in the evidence, as findings based on studies with important methodological limitations are likely to be downgraded. Future syntheses could include methodological limitations in a sampling framework. This could lead to higher confidence in some review findings. However, this approach could also potentially lead us to sample even fewer studies, which could have implications for other CERQual components, including our assessment of data adequacy or relevance. Another possible option is to identify findings that have been downgraded due to concerns about the methodological limitations of the contributing studies. Review authors could then choose to look at the pool of well conducted studies that have not been sampled to see if any include data that could contribute to the finding and could therefore be sampled into the synthesis. Further work is needed to explore the advantages and disadvantages of these different options.

A linked issue is that, to date, the best way in which to assess the methodological strengths and limitations of qualitative research is still contested [7, 24]. We believe that assessing the methodological strengths and limitations of included studies is feasible and is an important aspect of engaging with the primary studies included in a synthesis [24]. We would also argue that most readers make judgements about the methodological strengths and limitations of qualitative studies that they are looking at, and that the tools available to assess this help to make these judgements more transparent and systematic. To be useful, these judgements need to be linked to the synthesis findings, as part of a CERQual assessment of confidence in the evidence.

### Qualitative evidence synthesis updates

This type of purposive sampling could also be useful during synthesis updates. In this case, a review author could sample studies from the pool of included studies that would contribute to strengthening findings with very low or low confidence. Further work is needed to see how sampling processes and CERQual assessments impact on each other. In Table 6 we present different ways in which we believe different sampling methods could be used in future synthesis.

**Table 6:** Different types of sampling methods and ways of using them

Type of sampling [14]	Used or not used in this sampling framework	Potential ways in which we could use it in future
Extreme or deviant case sampling	We did not use this type of sampling in this review	This approach could be very useful when setting out sampling for a review when the review question addresses why something did or did not work or when re sampling after you have a set of findings i.e. when updating a review
Maximum variation sampling	Used to help create our sampling frame	We used this to create the first step of our sampling frame. It could also be useful to apply when sampling for other variables such as intervention, population or socioeconomic status.
Snowball or chain sampling	We did not use this type of sampling in this review	We believe that this approach could be used if there were key articles in the field. However, it should still be supported by a systematic search and sampling.
Theoretical or operational construct sampling	We did not use this type of sampling in this review	Useful if performing a systematic review of different theoretical or operational constructs. For example, theories of behaviour change as applied to different smoking prevention programs.
Criterion sampling	Used to help create our sampling frame	We decided on the criterion that were central to our research objective and created a sampling framework around them.
Stratified purposeful sampling	We did not use this type of sampling in this review	We have since used this approach in another review where we divided the studies by population and then applied the same sampling frame to each population.
Purposeful random sampling	We did not use this type of sampling in this review	We do not regard random sampling as useful or appropriate when trying to explore a specific phenomenon of interest
Combination or mixed purposeful sampling	Used to help create our sampling frame	We combined different sampling methods to create our three step sampling frame that worked for our specific research objective

In conducting the sampling for this synthesis and talking with other qualitative evidence synthesis authors it has become clear that more research and guidance are needed around this topic. Review authors need to try out different sampling methods and approaches and document the steps they took and how the sampling approach worked out. It would be useful to conduct research comparing different sampling approaches for the same synthesis question and looking at whether these result in different findings. Finally, it is important that better guidance is developed for review authors on how to apply different sampling approaches when conducting a qualitative evidence synthesis.

## Conclusions

We used purposive sampling to select 38 primary studies for the data synthesis using a three step-sampling frame. We employed a sampling strategy, as seventy-nine studies were eligible for inclusion in the synthesis. We feel that large numbers of studies can threaten the quality of the analysis in a qualitative evidence synthesis. We used the sampling strategy to decrease the number of studies to a manageable number.

Going forward, there is a need for research into purposive sampling for qualitative evidence synthesis to test the robustness of different sampling frameworks. More research also needs to be undertaken on how best to rate data richness within qualitative primary studies.

In conclusion, this systematic three-step approach to sampling may prove useful to other qualitative evidence synthesis authors. However, based on our experience it could be narrowed to a two-step approach with the combination of data richness and closeness to the synthesis objectives. Further steps could be added to address synthesis specific objectives such as population or intervention. As more syntheses are completed, the issue of sampling will arise more frequently and so approaches that are more explicit need to be developed. Transparent and tested approaches to sampling for synthesis of qualitative evidence are important to ensure the reliability and trustworthiness of synthesis findings.

## Additional files

Additional file 1: Overview of sampling stage and contribution to findings for primary studies included in the Qualitative Evidence Synthesis . This table presents an overview of each of the primary studies included in the qualitative evidence synthesis, the stage at which they were sampled and how many findings each study contributes to. (DOCX 13 kb)

Additional file 2: Study characteristics addressed in the CERQual concept of relevance. This table presents the different study charachteristics that can be addresses when applying the CERQual concept of relevance. (DOCX 16 kb)
