# Supplementary material for: Purposive sampling in a qualitative evidence synthesis: a worked example from a synthesis on parental perceptions of vaccination communication
Source: BMC Med Res Methodol. 2019 Jan 31;19:26. doi: 10.1186/s12874-019-0665-4 (PMC6357413; doi:10.1186/s12874-019-0665-4)
Supplement: Supplementary file 1 — Overview of sampling stage and contribution to findings for primary studies included in the Qualitative Evidence Synthesis . This table presents an overview of each of the primary studies included in the qualitative evidence synthesis, the stage at which they were sampled and how many findings each study contributes to. (DOCX 13 kb) [file 12874_2019_665_MOESM1_ESM.docx]

Additional file 1: Overview of sampling stage and contribution to findings for primary studies included in the Qualitative Evidence Synthesis

| **Study** | **Number of synthesis findings to which the study contributed** | **Sampling step** |
| --- | --- | --- |
| **Tadesse 2009** | **2** | **1** |
| **Babirye 2011** | **3** | **1** |
| **Henderson 2008** | **3** | **2** |
| **Figueiredo 2011** | **5** | **1** |
| **Kitayama 2014** | **6** | **2** |
| **Brunson 2015** | **6** | **3** |
| **Topuzoğlu 2007** | **7** | **1** |
| **Barbieri 2015** | **7** | **1** |
| **Bond 2011** | **7** | **2** |
| **Brunson 2013** | **7** | **3** |
| **Berhanel 2000** | **8** | **1** |
| **Hussain 2012** | **8** | **1** |
| **Harmsen 2012** | **9** | **2** |
| **Tomlinson 2013** | **9** | **2** |
| **Kowal 2015** | **9** | **2** |
| **Austin 2008** | **10** | **2** |
| **Brown 2014** | **10** | **2** |
| **Fowler 2007** | **11** | **1** |
| **Saada 2015** | **11** | **2** |
| **Blaisdell 2016** | **11** | **2** |
| **Gust 2008** | **12** | **2** |
| **Tickner 2010** | **12** | **2** |
| **Delkhosh 2014** | **13** | **1** |
| **Bond 1998** | **13** | **2** |
| **Shui 2005** | **13** | **2** |
| **Harmsen 2015** | **13** | **2** |
| **Dubé 2016** | **14** | **2** |
| **Benin 2006** | **14** | **3** |
| **Miller 2008** | **15** | **2** |
| **McMurray 2004** | **16** | **2** |
| **Sobo 2016** | **16** | **2** |
| **Hilton 2007** | **17** | **3** |
| **Evans 2001** | **18** | **2** |
| **Brown 2012** | **18** | **2** |
| **Fadda 2015** | **18** | **2** |
| **Tickner 2007** | **19** | **2** |
| **Guillaume 2004** | **20** | **2** |
| **Austvoll-Dahlgren 2010** | **22** | **3** |
| Sampling  step 1: Low and middle-income countries  Sampling step 2:  Data richness of 3-4 or higher  Sampling step 3: Closeness to the synthesis objectives | | |
