# Supplementary material for: Purposive sampling in a qualitative evidence synthesis: a worked example from a synthesis on parental perceptions of vaccination communication
Source: BMC Med Res Methodol. 2019 Jan 31;19:26. doi: 10.1186/s12874-019-0665-4 (PMC6357413; doi:10.1186/s12874-019-0665-4)
Supplement: Supplementary file 2 — Study characteristics addressed in the CERQual concept of relevance. This table presents the different study charachteristics that can be addresses when applying the CERQual concept of relevance. (DOCX 16 kb) [file 12874_2019_665_MOESM2_ESM.docx]

Additional file 2: Study characteristics addressed in the CERQual concept of relevance [25]

| **Micro context** | |
| --- | --- |
| *Population characteristics* | - Do particular characteristics related to the population specified in the review question (such as age, gender, socioeconomic status) raise concerns regarding the relevance of the review finding? - Is the population reported in sufficient detail to make comparisons? |
| *Characteristics of the setting and place* | - Do particular characteristics related to the setting or place as specified in the review question warrant concerns regarding relevance of the review finding (such as urban versus rural, private versus public, low income versus high income)? - Are the setting and place reported in sufficient detail to facilitate comparisons? |
| *Temporal characteristics* | - Are the data likely to be very different from the context specified in the review question because of when these data were collected? |
| **Meso-context** | |
| *Intervention characteristics* | - Do particular characteristics related to the intervention, such as who implemented it and how it was implemented, raise concerns regarding the relevance of the review finding to the review question? - Is the intervention reported in sufficient detail to make comparisons? |
| **Macro-context** | |
| *Policy or political issues, social climate, legislation* | - Do particular socio-political characteristics in the study setting, such as type of government, legality of the intervention, or social and cultural values, raise concerns regarding the relevance of the review finding to the review question? |
| **Cross cutting** | |
| *Phenomena of interest* | - Do particular characteristics, or lack of clarity, or lack of reporting concerning the phenomena of interest raise concerns regarding the relevance of the review finding to the question? |
